# Phenolic metabolites from *Ceratonia siliqua* L. pods: chemical characterization, soybean lipoxygenase-1 inhibition, and *in vitro* gallstone-dissolving capacity

**DOI:** 10.3389/fphar.2026.1945402

**Published:** 2026-09-30

**Authors:** Souheyla Mekraf, Mokhtaria Yasmina Boufadi, Fatiha Abbassene, Kamal Nedjari Benhadj Ali, Abdelkrim Chetibi, Soumia Keddari, Malika Meziane, Mohammed Bouzouina, Hamza Moussa, Zulzamri Salleh, Hichem Tahraoui, Jie Zhang, Abdeltif Amrane

**Affiliations:** 1 Laboratory of Bioeconomics, Food Safety, and Health, Department of Food Science, Faculty of Natural Sciences and Life, University of Abdelhamid Ibn Badis, Mostaganem, Algeria; 2 Faculty of Biological Sciences, University of Sciences and Technology Houari Boumediene (USTHB), Algiers, Algeria; 3 Laboratory of Sciences and Techniques of Valorization (STEVA), Faculty of Natural Sciences and Life, University of Abdelhamid Ibn Badis, Mostaganem, Algeria; 4 Laboratory of Plant Protection, Faculty of Natural Sciences and Life, University of Abdelhamid Ibn Badis, Mostaganem, Algeria; 5 Hospital Center University Beni Messous, Algiers, Algeria; 6 Research Laboratory for Crop Production and Protection in Chlef Region, Faculty of Natural Sciences and Life, University of Hassiba Benbouali, Chlef, Algeria; 7 Laboratoire de Biotechnologies et Protection des Ecosystèmes Agricoles et Naturels, Faculté des Sciences de la Nature et de la Vie et Sciences de la Terre, Université de Bouira, Bouira, Algeria; 8 Malaysian Institute of Marine Engineering Technology (UniKL MIMET), Universiti Kuala Lumpur, Lumut, Perak, Malaysia; 9 Laboratory of Biomaterials and Transport Phenomena (LBMTP), University Yahia Fares, Medea, Algeria; 10 Ecole Nationale Supérieure de Chimie de Rennes, University of Rennes, CNRS, Rennes, France; 11 School of Engineering, Newcastle University, Newcastle upon Tyne, United Kingdom

**Keywords:** *Ceratonia siliqua*, cholelithiasis, gallstone-dissolving capacity, HPLC/UV, in silico, soybean lipoxygenase-1

## Abstract

**Introduction:**

Carob (Ceratonia siliqua L., Fabaceae) is a Mediterranean tree whose pods are rich in phenolic metabolites and are credited with antioxidant, anti-inflammatory,hypolipidemic and antimicrobial properties.

**Methods:**

The chemical composition of MECp was determined by high-performance liquid chromatography coupled with ultraviolet detection (HPLC/UV). Free radical-scavenging and ferric-reducing capacities were measured using the 2,2-diphenyl-1-picrylhydrazyl (DPPH) and ferric-reducing antioxidant power (FRAP) analytical descriptors assays, and soybean LOX-1 inhibition was assessed in vitro and examined in silico by molecular docking against the enzyme (PDB ID: 3O8Y). Gallstones removed at surgery were confirmed as cholesterol stones by Fourier transform infrared spectroscopy with attenuated total reflectance (FTIR-ATR) and then incubated in MECp under continuous agitation for 4 weeks, with a sodium chloride solution (NaCl, 9 g/L) as control. Gallstone mass after drying at 40 °C, the percentage of gallstone-dissolving capacity (% GDC) and the pH of the medium were recorded weekly.

**Results:**

HPLC/UV analysis identified 20 phenolic metabolites in MECp. The extract contained 160.68 mg GAE/g DE of total phenolics and 9.19 mg QE/g DE of total flavonoids, with median inhibitory concentrations (IC50) of 0.16, 0.86 and 0.19 mg/mL for the DPPH, FRAP and soybean LOX-1 inhibition assays, respectively. Compared with the control, MECp produced a highly significant increase in % GDC over time, and the docking analysis revealed strong and stable interactions between the major polyphenols and the soybean LOX-1 active site.

**Discussion:**

The dissolution obtained with MECp is consistent with its phenolic composition, and the docking results agree with the soybean LOX-1 inhibition measured in vitro. These findings identify MECp as a promising natural candidate for cholesterol gallstone management and support further preclinical investigation.

## 1 Introduction

Gallstone sickness, also known as cholelithiasis, affects 15%–20% of the world population (Mikhaleva et al., 2021). Gallstones form mostly due to the dysregulated nucleation processes, accompanied by an excess of phospholipids and bile acids, which increase the cholesterol supersaturation in the bile, thereby promoting the development of this illness (Tazuma, 2017; Sharma and Sharma, 2022) and may result in clinical complications such as biliary obstruction, cholangitis, and pancreatitis (Rajendran et al., 2025). Based on cholesterol levels, gallstones are categorized into three groups: cholesterol (>70%), pigment (≥30%) (Tazuma, 2017), and mixed (30%–70%) (Mashina et al., 2020). Genetics, advanced age, female sex, obesity, diabetes, high cholesterol concentration, and physical inactivity are all linked risk factors for this condition, which commonly requires urgent surgery (Akmal et al., 2022; Hendarto et al., 2023; Mi et al., 2024). Delay in treatment and the presence of comorbidities may increase mortality. Regular monitoring of at-risk groups is essential for early identification and prevention of catastrophic consequences (Mikhaleva et al., 2021). Cholelithiasis is a frequent hepatobiliary condition for which non-surgical therapeutic strategies are inadequate. Botanical drug may be favored over synthetic medications due to their richness in synergistic metabolites and fewer side effects, offering different therapeutic benefits such as lipid-lowering, anti-inflammatory, and choleretic properties (Vo et al., 2023; Ubhenin et al., 2023; Amelia et al., 2024; Rajendran et al., 2025).

Among such plant species, the carob tree (*Ceratonia siliqua* L.), is a member of the Fabaceae (Leguminosae) family. It is a perennial tree from the Mediterranean area (Micheli et al., 2024). Carob components offer a cost-effective alternative for enriching fortified foods (Aydın and Özdemir, 2017), owing to their valuable nutritional profile and wide range of therapeutic properties, involving relief from digestive issues (Senouci et al., 2019), management of urinary ailments, anemia, and sexual dysfunctions (Uzun and Koca, 2020), and exhibiting antidiabetic, anti-inflammatory, antimicrobial, antiulcer, and cancer-fighting benefits (Darwish et al., 2021; Gregoriou et al., 2021; Elaoufi et al., 2022).

In this work, a multidisciplinary study of the methanolic extract of *C. siliqua* pods was conducted to explore its phytochemical and biological properties. The main objective was to characterize the phenolic metabolites present in the extract and to evaluate their therapeutic potential through various experimental and theoretical approaches. First, an extraction step was performed on the carob pods. The samples were collected, cleaned, air-dried, and then ground into a powder. The extraction of polyphenols was carried out by maceration in methanol, followed by evaporation of the solvent to obtain the crude extract used for the analyses. Next, a detailed chemical characterization of the extract was performed. HPLC/UV analysis allowed for the identification and quantification of several phenolics and flavonoids. The content of these metabolites were determined using the Folin–Ciocalteu and aluminum chloride methods, respectively. The extract was evaluated for its free radical-scavenging and ferric reducing capacities using the DPPH and FRAP analytical descriptors assays, while soybean lipoxygenase-1 inhibition was evaluated to determine whether the extract modulates an enzyme involved in inflammatory processes. Finally, an *in vitro* gallstone dissolution study was conducted to assess its litholytic potential. Cholesterol gallstones were incubated with the extract under controlled conditions for several weeks, and their mass loss and pH changes were monitored to evaluate the extract’s gallstone dissolution capacity. Finally, an *in silico* molecular docking analysis was performed to study the interactions between the main identified phenolic metabolites and the active site of soybean LOX-1. The results were statistically analyzed to ensure the reliability and significance of the data obtained.

This study provides a novel and integrated investigation of the methanolic extract of *C. siliqua* pods (MECp), combining phytochemical profiling, *in vitro* biological assay, and *in silico* molecular docking. Although no ethnobotanical evidence has documented the use of *C*. *siliqua* for gallstone treatment, this study presents the first report evaluating the gallstone-dissolving capacity of *C. siliqua* pod extract under controlled *in vitro* conditions, where complete cholesterol gallstone dissolution was achieved within 4 weeks of treatment. In addition, this work offers a comprehensive characterization of MECp using HPLC/UV analysis, identifying a diverse phenolic profile, and linking this composition to its important antioxidant characteristics and soybean lipoxygenase-1 inhibition. The study further integrates molecular docking simulations targeting soybean LOX-1, providing mechanistic insight into the observed bioactivities and supporting the potential therapeutic relevance of the identified phenolics. Overall, the novelty lies in the combined demonstration of antioxidant, enzyme inhibitory, and gallstone dissolution capacity of Algerian *C. siliqua* pod extract, supported by both experimental and computational approaches, which has not been previously reported in an integrated manner.

## 2 Materials and methods

### 2.1 Reagents

All chemicals were purchased from Sigma-Aldrich (St. Louis, MO, United States), such as absolute methanol (CH_4_O, ≥99.7%), 2,2-diphenyl-1-picrylhydrazyl (DPPH, C_18_H_12_N_5_O_6_), 2,4,6-tripyridyl-s-triazine (TPTZ, C_18_H_12_N_6_), 6-Hydroxy-2,5,7,8-tetramethylchroman-2-carboxylic acid (Trolox, C_14_H_18_O_4_), soybean lipoxygenase, Type I-B, from Glycine max (Soybean LOX-1, 8000 U/mL, L7395 catalogue number), linoleic acid (C_18_H_32_O_2_), formic acid (CH_2_O_2_, 98%–100%), trifluoroacetic acid (TFA, C_2_HF_3_O_2_, 99%), boric acid (H_3_BO_3_), acetic acid (C_2_H_4_O_2_), hydrochloric acid (HCl), acetonitrile (C_2_H_3_N), sodium acetate (C_2_H_3_NaO_2_), potassium chloride (KCl), disodium hydrogen phosphate (Na_2_HPO_4_), potassium phosphate monobasic dihydrate (KH_2_PO_4_), and certified phenolic standards (trans-cinnamic (C_9_H_8_O_2_), gallic (C_7_H_6_O_5_), syringic (C_9_H_10_O_5_), sinapic (C_11_H_12_O_5_), quinic (C_7_H_12_O_6_), protocatechuic (C_7_H_6_O_4_), ferulic (C_10_H_10_O_4_), *p*-coumaric (C_9_H_8_O_3_), caffeic (C_9_H_8_O_4_), carnosic (C_20_H_28_O_4_), chlorogenic (C_16_H_18_O_9_), vanillic (C_8_H_8_O_4_), ellagic (C_14_H_6_O_8_), ascorbic (C_6_H_8_O_6_), tannic (C_76_H_52_O_46_) acids, catechin (C_15_H_14_O_6_), epicatechin (C_15_H_14_O_6_), catechol (C_6_H_6_O_2_), carnosol (C_20_H_26_O_4_), thymol (C_10_H_14_O), galangin (C_15_H_10_O_5_), pinocembrin (C_15_H_12_O_4_), rutin (C_27_H_30_O_16_), chrysin (C_15_H_10_O_4_), luteolin (C_15_H_10_O_6_), fisetin (C_15_H_10_O_6_), myricetin (C_15_H_10_O_8_), naringin (C_27_H_32_O_14_), acacetin (C_16_H_12_O_5_), kaempferol (C_15_H_10_O_6_), quercetin (C_15_H_10_O_7_), menthol (C_10_H_20_O), and bis-methylated quercetin (C_15_H_10_O_7_), Folin-Ciocalteu’s Reagent (FCR), aluminum chloride (AlCl_3_), ferric chloride (FeCl_3_), sodium carbonate (Na_2_CO_3_), sodium hydroxide (NaOH), and sodium chloride (NaCl). This study exclusively utilized high-quality analytic reagents and chemicals. Only high-quality analytical-grade reagents and chemicals were employed throughout this study.

### 2.2 Plant material collection and extraction

The mature pods of *C. siliqua* L. were randomly harvested in May 2024 from the Tenes region in Algeria (36°30′44″N, 1°18′16″E; latitude 36.5122, longitude 1.30444; altitude 27 m). The collected material was botanically identified by Prof. Mohammed Bouzouina (Faculty of Natural and Life Sciences, University of Mostaganem). The Laboratory of Plant Protection, Abdelhamid Ibn Badis University (Mostaganem, Algeria) received 12052024LPV, a reference voucher specimen. The seedless pods were thoroughly cleaned by water, air-dried under the shade, and finely pulverized. The powder was sealed until usage. For the extraction, 20 g of *C. siliqua* pod powder was macerated in 200 mL of methanol (MeOH, 99.7%) for 72 h. Rotary evaporation (BOECO, type RVO 400 SD, Germany) yielded carob pod methanolic extract. It was kept at 4 °C until required (Imene Reffas et al., 2026).

### 2.3 Analysis of *C. siliqua* extract by HPLC/UV

The separation of MECp was performed using an Agilent 1100 high-performance liquid chromatography equipment (LC-2040C, Shimadzu, Japan). The separation was performed using an Agilent 120 EC Poroshell column (100 mm × 2.1 mm, 2.7 µm) with a mobile phase consisting of water/trifluoroacetic acid/formic acid (99:0.25:0.75) (A) and acetonitrile (B). A 10 µL injection volume was used to perform elution at 55 °C with a flow rate of 0.6 mL/min. The solvent gradient protocol was as outlined: (0 min, 0% B) (1 min, 10% B), (2 min, 12.5% B) (3 min, 15% B), (9 min, 80% B) (10 min, 100% B), (11 min, 100% B), and (14 min, 0% B). UV detection was performed at 270 nm and 320 nm after 5 min. The extract was diluted 1:100 (v/v) in methanol for sample preparation. Identification of compounds was based on retention times (Rt) and spectra corresponding with 34 phenolic standards, including phenolic acids (trans-cinnamic acid, gallic acid, syringic acid, sinapic acid, quinic acid, protocatechuic acid, ferulic acid, *p*-coumaric acid, caffeic acid, prenyl caffeate acid, carnosic acid, chlorogenic acid, vanillic acid, and ellagic acid), flavonoids (catechin, epicatechin, catechol, carnosol, thymol, galangin, pinocembrin, rutin, chrysin, luteolin, fisetin, myricetin, naringin, acacetin, kaempferol, and quercetin), tannins (tannic acid), and other compounds (ascorbic acid, menthol, and bis-methylated quercetin). Methanol solution stocks (1 mg/mL) of each standard were made. The phenolic components were determined in mg per Gram of crude extract using reference compound calibration curves (Chaa et al., 2019).

### 2.4 Phytochemical characterisation of MECp

#### 2.4.1 Phenolic contents determination

The Folin-Ciocalteu method estimated the total phenolic content (TPC) with some modifications (Gadouche et al., 2025). Incubate 125 µL extract, 500 μL distilled water, and 125 μL 10% Folin-Ciocalteu’s reagent (FCR) at room temperature for 5 min. Next, add 1250 μL of 7% Na_2_CO_3_ solution and 1000 μL of distilled water. Following 90 min at ambient temperature, a UV–Vis spectrophotometer (Optizen Pop, Korea) detected optical density at 765 nm against a blank sample. The calibration curve was set up using gallic acid. The outcomes were determined as milligrams of gallic acid equivalents per Gram of dry extract (mg GAE/g DE). Every measurement was done three times. The total flavonoid content (TFC) of MECp was quantified using a colorimetric method with aluminum chloride (Guenane et al., 2024). The experiment involved incubating 1000 µL of 2% AlCl_3_ solution and 1000 µL of extract at ambient temperature for 60 min. The absorbance was obtained at 517 nm using a UV-Vis spectrophotometer, with quercetin as a reference. Results were reported as mg QE/g DE, with triple measurements.

#### 2.4.2 DPPH and FRAP analytical descriptors assays

##### 2.4.2.1 DPPH assay

For this assay, 250 µL of *C. siliqua* extract (1, 0.5, 0.25, 0.125, and 0.0625 mg/mL) were mixed with 1000 µL of 0.004% DPPH solution. Following 30 min in the dark at ambient temperature, an absorbance was read at 517 nm compared to a blank. Trolox was a control. The radical inhibition percentage (% I) was estimated applying Equation 1 (Chantan et al., 2023; Simanjuntak et al., 2024).

$$I\;\left(\%\right)=\left[\frac{A_{\text{control}}-A_{\text{sample}}}{A_{\text{control}}}\right]\times 100$$ (1)

where A_Control_ represents the absorbance of the control (DPPH solution without extract), and A_Sample_ represents the absorbance of the sample or standard. The median inhibitory concentration (IC_50_) was determined using a linear regression model. Every measurement was done three times.

##### 2.4.2.2 FRAP assay

The phenolic metabolites in *C. siliqua* extract were tested for ferric-reducing antioxidant power (FRAP) assay using a previously reported technique (Nedjari Benhadj Ali et al., 2025), with slight adjustments. The sodium acetate (C_2_H_3_NaO_2_) buffer (pH 3.6, 300 mM, prepared in 0.28 mM acetic acid), ferric chloride (FeCl_3_, 20 mM), and a 2,4,6-tripyridyl-s-triazine (TPTZ) solution (10 mM prepared in 40 mM HCl) in a 10:1:1 ratio (v/v/v) constituted a FRAP reagent solution, followed by an incubation period at 37 °C for 10 min. The combination included 9 mL FRAP solution, 300 μL *C. siliqua* pod extract (1, 0.5, 0.25, 0.125, and 0.0625 mg/mL), and 300 µL distilled water. Versus a blank sample, the absorbance was measured at 593 nm after 30 min at ambient temperature. Equation 1 was used to calculate the radical inhibition percentage, and the IC_50_ was then determined. The Trolox was employed as a standard positive control. Every measurement was done three times.

### 2.5 *In vitro* assessment of soybean lipoxygenase-1 inhibition

The soybean lipoxygenase-1 inhibition test was conducted in accordance with the method outlined by Gunathilake et al. (2018). In brief, add 1 mL of sodium borate buffer (0.1 M, pH 8.8) and 10 µL of soybean LOX-1 and incubate at ambient temperature for 5 min. Introduce 10 µL of 10 mM linoleic acid substrate to start the reaction. After adding 1 mL of sodium borate buffer, absorbance was measured at 234 nm. A control phosphate buffer solution without extract was used for calculating soybean LOX-1 inhibition (% I) applying Equation 2. In mg/mL, the IC_50_ was reported. Every measurement was done three times.

$$I\;\left(\%\right)=\left[\frac{A_{\text{control}}-A_{\text{sample}}}{A_{\text{control}}}\right]\times 100$$ (2)

### 2.6 Assessment of gallstone-dissolving capacity *in vitro*

#### 2.6.1 Gallstones

The gallstones were surgically removed from an adult patient with cholelithiasis by the General Surgery Service at Bourdj Bounaama Hospital (Tissemsilt, Algeria). Immediately after surgery, gallstones were washed with NaCl solution (9 g/L) and kept in sterile bottles at 4 °C. Six gallstones were used for the dissolution testing. Their composition was analyzed using FTIR-ATR (IR Affinity-1S, Shimadzu, Japan). All procedures received approval from the local ethics committee.

#### 2.6.2 Gallstone-dissolving capacity

The previously reported approach was adopted to assess MECp’s gallstone-dissolving cholesterol capability *in vitro* (Mekraf et al., 2025). To avoid mechanical damage, each gallstone was individually wrapped in sterile permeable gauze and placed in a glass Erlenmeyer flask containing 100 mL of MECp (1 mg/mL in 9 g/L NaCl) or the control solution (NaCl, 9 g/L). The flasks were hermetically sealed and agitated at 130 rpm at ambient temperature for 9 weeks, corresponding to the conventional duration of medical treatment. Each experimental group consisted of three flasks, with one gallstone per flask. The mean initial gallstone mass was 209.33 ± 1.53 mg for the MECp group and 207.00 ± 2.00 mg for the saline control group. To assess weight reduction, gallstones were removed weekly from the reaction solutions, dried at 40 °C for 18 h, and weighed using a precision balance (Ohaus PA213, China). The percentage of gallstone dissolution (% GDC) was calculated using Equation 3. All measurements were performed in triplicate.

$$\text{GDC}\left(\%\right)=\left[\frac{w_{i}-w_{f}}{w_{i}}\right]\times 100$$ (3)

where w_i_ represents the initial weight (mg) of the gallstone and w_f_ is the final weight (mg) after incubation.

#### 2.6.3 Progression of pH

The pH of the testing solution was measured initially and then weekly to monitor its progression using a pH meter (HANNA HI 2211, Romania). Every measurement was done three times.

### 2.7 *In silico* studies of soybean lipoxygenase-1

The three-dimensional structure of soybean lipoxygenase-1 (soybean LOX-1, PDB ID: 3O8Y) was obtained from the Protein Data Bank (RCSB PDB) on 20 January 2026. Protein preparation was carried out using BIOVIA Discovery Studio Visualizer 2020, which removed co-crystallized ligands, water molecules, and heteroatoms before storing the cleaned structure in PDB format. The phenolic metabolites found in the methanolic extract of mature carob pods (MECp) were retrieved from the PubChem database in SDF format and translated to PDB format using BIOVIA Discovery Studio Visualizer 2020. Molecular docking was conducted using AutoDock Vina 1.1.2, employing a blind docking method that covered the full protein surface. The exhaustiveness parameter was set at 8, and docking calculations were performed using flexible ligand conformations in PDBQT format. The best protein–ligand complexes were selected according to binding affinity values (kcal/mol). Interaction profiles and docking conformations of the top-ranked phenolic metabolites were analyzed and visualized using BIOVIA Discovery Studio Visualizer 2020 (Moussa et al., 2025).

### 2.8 Data processing and statistical analysis

Results are shown as mean ± SD, where each experiment was repeated three times. Statistics were analyzed using Past 4.03 (2020) and IBM SPSS Statistics 22 (2023). Data were examined using Tukey’s *post hoc* test after a one-way ANOVA. At *P < 0.05*, differences were considered significant; at *P < 0.01* and *P < 0.001*, highly significant; and at *P > 0.05*, not significant. It was compared to the NaCl control. Graphs were prepared using GraphPad Prism 10.3.1 (509).

## 3 Results and discussion

### 3.1 HPLC-UV analysis of MECp

MECp underwent HPLC/UV analysis, and the chromatograms were recorded at 270 and 320 nm (Figure 1a,b, respectively). Table 1 displays MECp’s phenolic acids and flavonoids. Caffeic acid (6.94 mg/g, retention time 3.879 min) is the predominant phenolic acid, while quercetin and chrysin are the principal flavonoids (14.98 mg/g, 1.719 min, and 11.65 mg/g, 2.188 min, respectively). In addition, syringic, tannic, ellagic, and carnosic acids are found in negligible amounts in the MECp extract.

**FIGURE 1:**
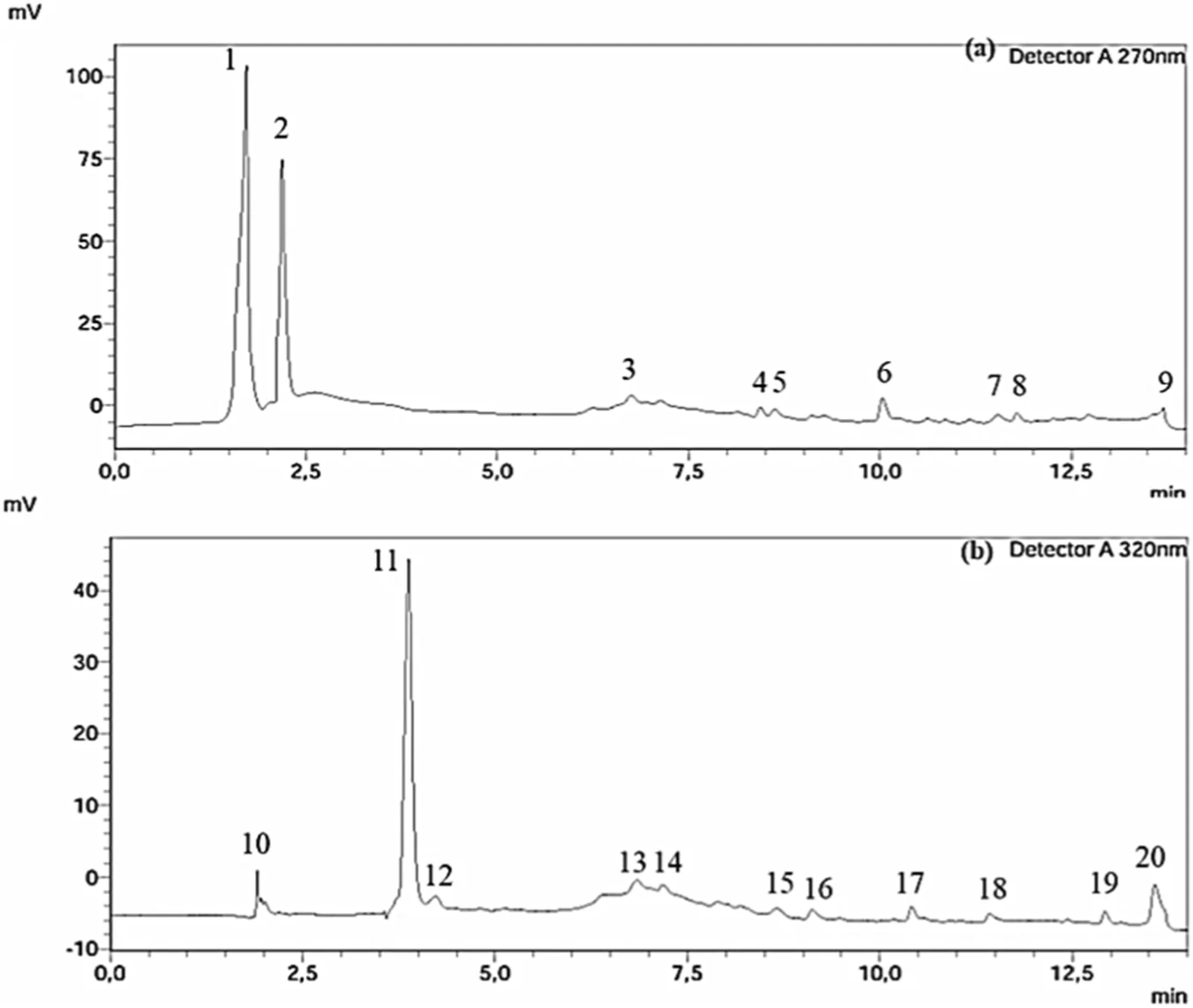
Chromatograms of MECp at 270 **(a)** and 320 nm **(b)** from HPLC/UV: peaks: 1: quercetin; 2: chrysin; 3: kaempferol; 4: catechin; 5: epicatechin: 6: pinocembrin; 7: luteolin; 8: myricetin; 9: thymol; 10: ascorbic acid; 11: caffeic acid; 12: prenyl caffeate acid; 13: gallic acid; 14: ferulic acid; 15: tannic acid; 16: ellagic acid; 17: trans-cinnamic acid; 18: syringic acid; 19: carnosic acid; 20: *p*-coumaric acid.

**TABLE 1:** HPLC/UV methanolic carob pod extract (MECp) composition (mg/g).

Peak number	Components	Level (mg/g MECp)	Rt _MECp_ (min)
1	Quercetin	14.98	1.719
2	Chrysin	11.65	2.188
3	Kaempferol	1.56	6.751
4	Catechin	1.32	8.442
5	Epicatechin	1.31	8.623
6	Pinocembrin	1.85	9.811
7	Luteolin	1.13	11.167
8	Myricetin	1.11	11.544
9	Thymol	1.19	13.449
10	Ascorbic acid	0.32	1.909
11	Caffeic acid	6.94	3.879
12	Prenyl caffeate acid	0.19	4.231
13	Gallic acid	0.21	6.85
14	Ferulic acid	0.20	7.181
15	Tannic acid	0.11	8.66
16	Ellagic acid	0.11	9.12
17	Trans-cinnamic acid	0.14	10.181
18	Syringic acid	0.09	11.426
19	Carnosic acid	0.11	12.925
20	*P*-coumaric acid	0.30	13.575

MECp: methanolic extract of carob pods, Rt _MECp_: retention time of MECp.

Previous investigations have shown varied phenolic metabolites in MECp extract. The composition comprises phenolic acids like ferulic, rosmarinic, and caffeic acids, flavonoids, including kaempferol, chrysin, galangin, pinocembrin, quercetin, luteolin, myricetin, and genistein, as well as other notable compounds. Ferulic acid was identified as the predominant phenolic acid (7.4 mg/g, 2.38 min), whereas kaempferol represented the major flavonoid (3.12 mg/g, 3.87 min), as reported by Elaoufi et al. (2022).

Furthermore, HPLC/UV analysis of Egyptian *C. siliqua* pods enabled the identification of several key phenolics, with kaempferol, gallic acid, quercetin rhamnoside, polydatin, fraxidin, and cinnamic acid derivatives being the most abundant (Al-Olayan et al., 2016). In addition, this species is considered an important source of polyphenols (tannic and gallic acids) and flavonoids (quercetin and myricetin), which supports earlier findings published by Dahmani et al. (2023) and Micheli et al. (2024). These results further confirm the phytochemical richness of *C. siliqua* pods and corroborate previously published data. The variability of experimental techniques limits the comparison of this study’s findings with those in the literature. Phenolic profiles vary according to extraction procedures, plant species, and sample maturity (Benchikh and Louailèche, 2014). These secondary metabolites exhibit several beneficial attributes, notably antioxidant, analgesic, antibacterial, anti-inflammatory, hepatoprotective, and neuroprotective. Several polyphenols, especially phenolic acids like sinapic acid, regulate cholesterol metabolism and gallstone dissolusion capacity (Chambers et al., 2019; Toma et al., 2020; Swarne et al., 2021; Rajendran et al., 2025).

### 3.2 Phytochemical characterisation of MECp

#### 3.2.1 Contents of total phenolic and flavonoid

The methanolic extract of *C. siliqua* pods contained 160.68 mg GAE/g and 9.19 mg QE/g ED of phenolics and flavonoids, respectively (Table 2).

**TABLE 2:** Total phenolic compounds, *in vitro* DPPH, FRAP, and soybean LOX-1 inhibitory tests of the MECp**.**

Sample	TPC (mg GAE/g DE)	TFC (mg QE/g DE)	DPPH IC_50_ (mg/mL)	FRAP IC_50_ (mg/mL)	Soybean LOX-1 inhibitory IC_50_ (mg/mL)
MECp	160.68 ± 2.55	9.19 ± 0.16	0.16 ± 0.00	0.86 ± 0.03	0.19 ± 0.11
Trolox	-	-	0.01 ± 0.00	0.05 ± 0.00	-

Test outcomes are presented as mean ± standard deviation from three tests. -: absent. The values are expressed as ± mean SD (n = 3).

In contrast, Al-Olayan et al. (2016) discovered a lower polyphenol amount of 68.3 mg GAE/mg of dried pod and a similar flavonoid level of 9.8 mg QE/mg of the same species’ methanolic pod extract from Egypt. Another study on the same species in Yemen found that the methanolic pod extract had a lower polyphenol content, about 127.02 mg GAE/mg DE, while the flavonoid level was higher, reaching 49.74 mg QE/mg DE (Qasem et al., 2018). The same species’ pod extract, Tunisian, contains higher amounts of polyphenols, with values of 465.50 mg GAE/g DE and 24.60 mg QE/g DE (Hsouna et al., 2012). Factors such as the plant species, geographic origin, harvest season, morphological and ecological traits, plant parts used, drying and storage methods, developmental stage, and the conditions and techniques employed during extraction might affect the variation in phytochemical levels (Ćujić et al., 2016).

#### 3.2.2 Free radical-scavenging and ferric-reducing descriptors of MECp

The MECp exhibited an IC_50_ of 0.16 mg/mL in the DPPH assay and 0.86 mg/mL in the FRAP assay. Trolox, used as the reference standard, showed lower IC_50_ values than the extract (0.01 mg/mL and 0.05 mg/mL for the DPPH and FRAP assays, respectively), indicating greater free-radical scavenging and ferric ion reduction (Table 2).

However, this disparity does not diminish the plant’s usefulness as a safe naturally occurring antioxidant source, unlike synthetic anti-radical compounds, which are associated with toxicity risks (Nedjari Benhadj Ali et al., 2025). A lower DPPH IC_50_ value (22.24 mg/mL) was reported for the methanolic extract of *C. siliqua* pods compared to the present findings (Dammak et al., 2024). Conversely, Qasem et al. (2018) displayed a slightly greater free-radical-scavenging in the same species (IC_50_ = 0.011 mg/mL). MECp may neutralize reactive oxygen species and inhibit myeloperoxidase, thereby reducing oxidative damage (Rtibi et al., 2015; Rtibi et al., 2017). The reduction of free radicals is attributed to phenolic metabolites, which inhibit free radical chain reactions by hydrogen atom donation. Accordingly, the polyphenols of *C. siliqua* may contribute to the interruption of oxidative chain reactions (Murugan and Parimelazhagan, 2013).

### 3.3 Soybean lipoxygenase-1 inhibition assay

The findings presented in Table 2 describe the inhibition of soybean lipoxygenase-1 by the *C. siliqua* extract.

MECp effectively suppressed soybean LOX-1, with an IC_50_ of 0.19 mg/mL. Jamous et al. (2018) showed that the leaf of the same species methanolic extract had a lower soybean lipoxygenase-1 inhibition (IC_50_ = 0.8 mg/mL) compared to the results of the present study.

### 3.4 Gallstone-dissolving capacity *in vitro*

#### 3.4.1 Gallstone characterization

The FTIR-ATR spectrum of the cholesterol gallstones is presented in Figure 2. The spectrum was acquired over the range of 3,000–750 cm^-1^.

**FIGURE 2:**
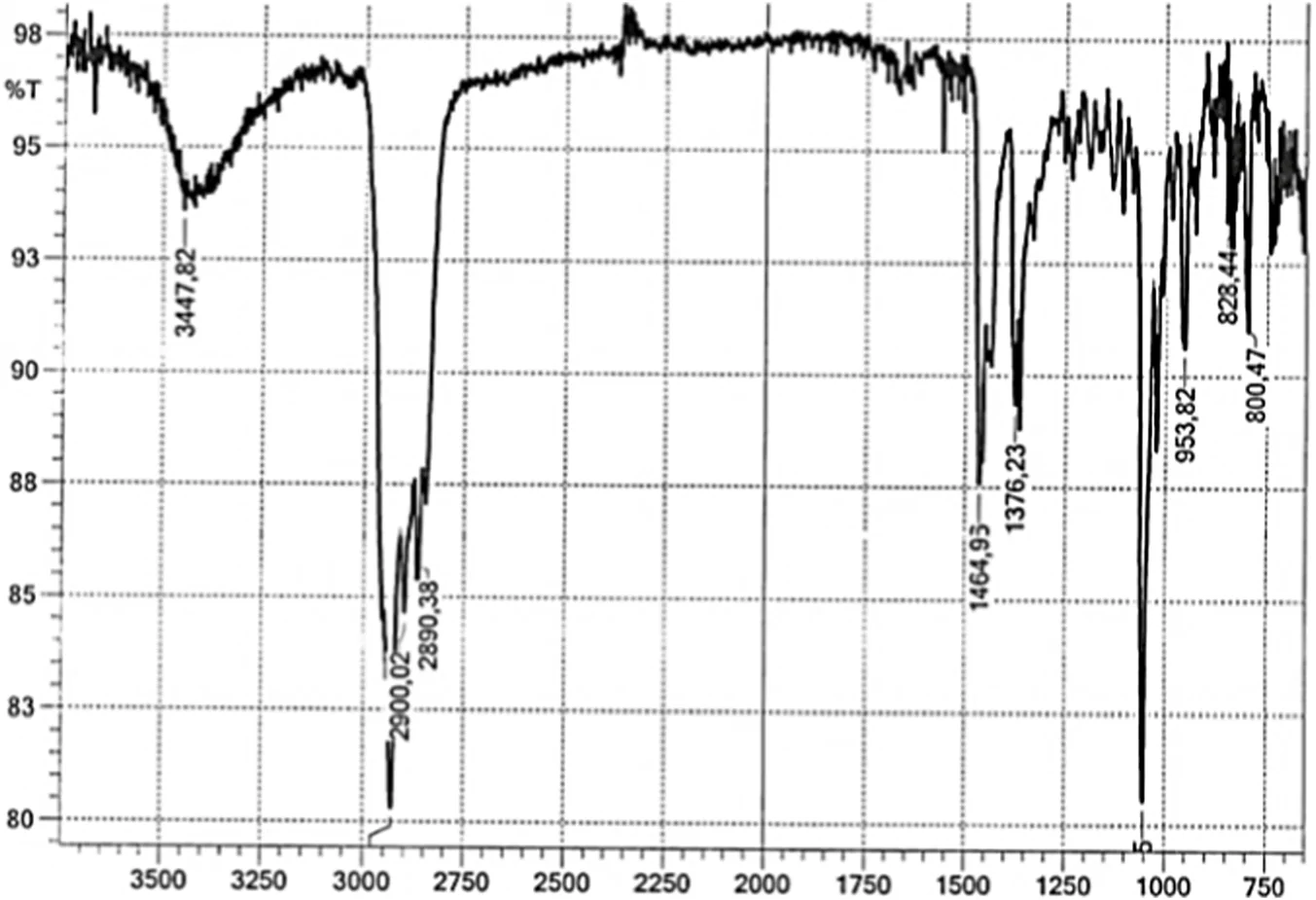
Fourier transform infrared (FTIR) spectroscopy graph showing percent transmittance versus wavenumber in inverse centimetres, plotted from about 3,700 to 700 cm^−1^.

#### 3.4.2 Effect of MECp in gallstone-dissolving capacity

Over 4 weeks, MECp dissolved cholesterol gallstones *in vitro* (Figure 3). The initial mass of gallstones was 209.33 ± 1.53 mg for the methanolic extract of *C. siliqua* and 207 ± 2.00 mg for the NaCl control. By the fourth week (W4) of MECp treatment, gallstones were completely dissolved. In contrast, the gallstone-dissolving capacity of the NaCl control showed only 2.41% ± 0.46% dissolution. All treatment conditions demonstrated a substantially greater reduction (*P < 0.001*) in gallstone mass over time. The investigation found that *C. siliqua* pod highly significantly enhanced (*P < 0.001*) cholesterol gallstone dissolution as opposed to control treatment throughout the research. The noted dissolution may result from the polyphenols of the studied extract.

**FIGURE 3:**
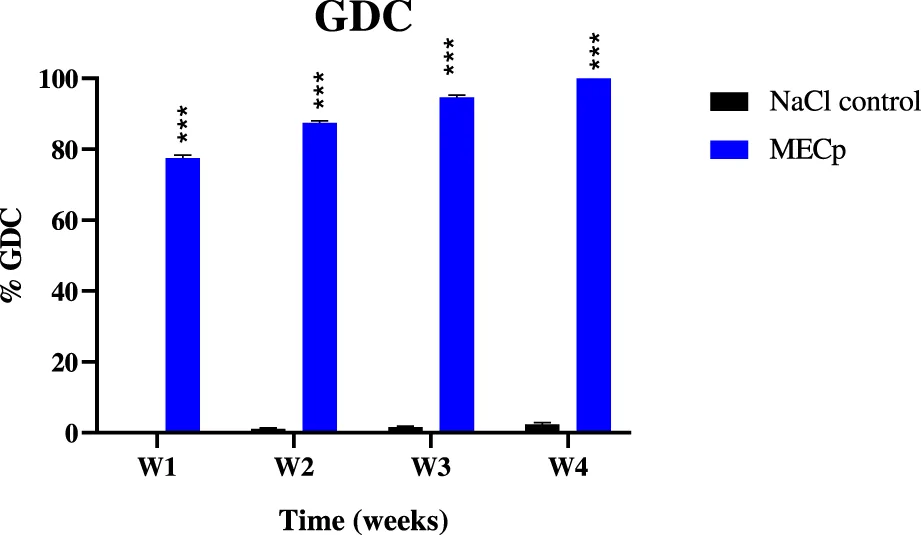
Development of MECp cholesterol gallstone-dissolving capacity (% GDC) over time: MECp: carob pod methanolic extract; NaCl control: sodium chloride. Results of three experiments are displayed as mean ± standard deviation. ***P < 0.001 versus control.

These findings suggest that their composition, abundance, and interacting actions might impact the level of mass loss (Amoura et al., 2018). In this context, no publicly available findings exist about the *C. siliqua* extract pods. In *Bunium incrassatum*, the methanolic root extract (MERO) and the methanolic seed extract (MESE) achieved complete dissolution of cholesterol gallstones within five and 9 weeks, respectively. The methanolic stem extract (MEST) dissolved 97.58% of the gallstones (Mekraf et al., 2025). According to Chekroune and Benamara (2017), after 2 weeks of therapy, the percentage of GDC was 72% for *Citrus limon* juice and 26% for *Herniaria hirsuta* extracts. Under the same testing circumstances, the *C. siliqua* extract demonstrated much greater gallstone-dissolving capacity. These findings suggest that their composition, abundance, and synergistic interactions may cause mass loss (Amoura et al., 2018). In this regard, there are no publicly accessible results on *C. siliqua* extract pods. In *B. incrassatum*, MERO and MESE achieved complete dissolution of cholesterol gallstones within five and 9 weeks, respectively, while MEST dissolved 97.58% of gallstones (Mekraf et al., 2025). According to Chekroune and Benamara (2017), after 2 weeks of treatment, the percentage of gallstone dissolution was 72% for *C. limon* juice and 26% for *H. hirsuta* extracts. Under the same experimental conditions, the *C. siliqua* extract demonstrated a markedly greater gallstone-dissolving capacity.

Tannic acid modulates lipid metabolism by inhibiting soybean lipoxygenase-1, thereby reducing triglyceride hydrolysis and cholesterol esterification. This effect decreases cholesterol micelle absorption and cholesterol crystallization, which helps prevent cholelithiasis and regulate blood cholesterol levels. Structure, especially the number and location of hydroxyl groups, facilitates non-covalent soybean LOX-1 active site interactions, making phenolic metabolites effective. Consequently, their consumption may exert beneficial effects on metabolic function, notably in preventing dyslipidemia and obesity. Furthermore, caffeic and ferulic acids may correct inactive liver progenitor cells by activating the hepatic Farnesoid X Receptor (FXR), which stimulates subsequent nuclear responses, including the Pregnane X Receptor (PXR), thereby enhancing bile acid synthesis and reducing biliary cholesterol saturation (Moreno-Córdova et al., 2020). Phenolic acids act through a second route. Caffeic, ferulic, and sinapic acids upregulate cholesterol 7α-hydroxylase (CYP7A1), the rate-limiting enzyme of bile acid synthesis, and adjust the hepatic ABCG5/ABCG8 sterol transporters through the LXRα pathway. Both effects lower the biliary cholesterol saturation index, which helps dissolve cholesterol-rich gallstones (Chambers et al., 2019; Rajendran et al., 2025).

#### 3.4.3 pH progression

Figure 4 shows the pH change during the gallstone-dissolving capacity assessment. The tested pod extract and the NaCl control had initial pH values of 7.89 ± 0.00 and 6.48 ± 0.00, respectively. After 4 weeks, the pH of the gallstone medium decreased to 7.13 ± 0.06 for MECp and to 6.11 ± 0.02 for the saline control. All treated groups exhibited a significantly greater reduction in pH over time (*P < 0.001*). However, the pH values of the plant extract-treated samples were largely identical to the control group’s NaCl values.

**FIGURE 4:**
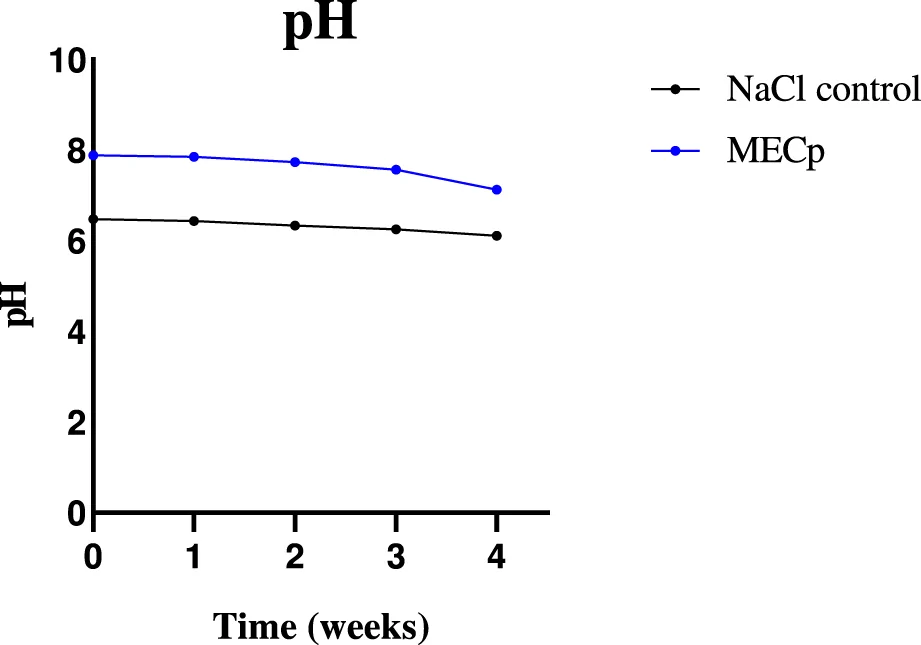
Time-dependent pH evolution in MECp with cholesterol gallstone weight groups: MECp: carob pod methanolic extract; NaCl control: sodium chloride. Results of three trials appear as mean ± standard deviation. The decrease in pH over time was significant in both groups (P < 0.001).

Recent data imply that the pH of these samples does not have a significant impact. The pH of these samples does not have a notable impact on cholesterol gallstone dissolution (Amoura et al., 2018; Mekraf et al., 2025). These findings confirmed previous reports.

### 3.5 Molecular docking

Hierarchical clustering analysis revealed a clear distribution of the investigated phenolic compounds according to their docking affinities toward soybean lipoxygenase-1. The docking energies ranged from approximately −5.90 to −8.90 kcal/mol, indicating moderate to strong interactions with the enzyme active site. Phenolic metabolites presenting the most negative binding energies were grouped together, reflecting similar interaction patterns and higher predicted inhibition against soybean LOX-1. Notably, the eight phenolic compounds illustrated in Figures 5–7, namely, ellagic acid, catechin, chrysin, quercetin, pinocembrin, kaempferol, myricetin, and luteolin, exhibited the strongest binding affinities toward the enzyme, suggesting enhanced stability within the catalytic pocket.

**FIGURE 5:**
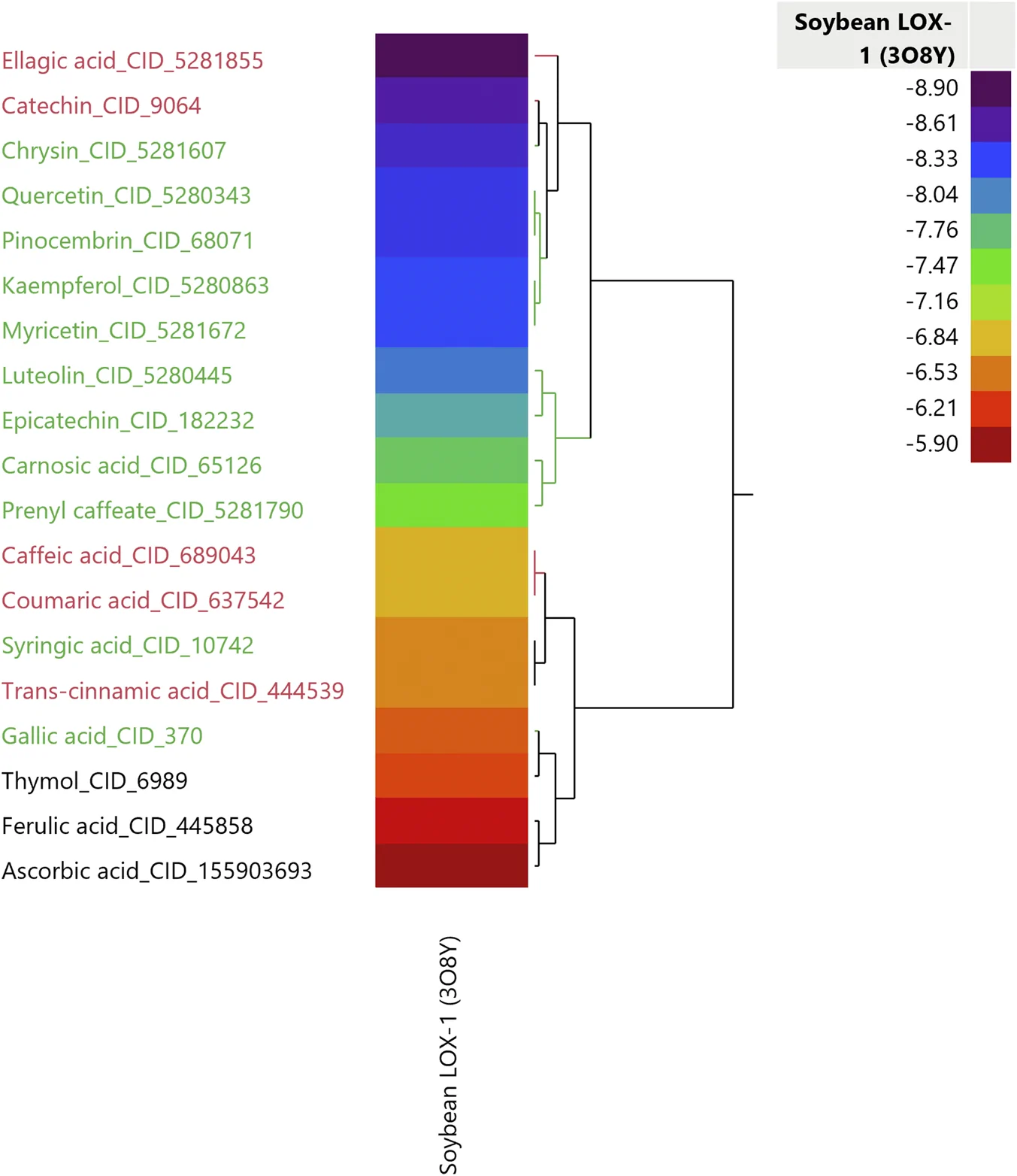
Cluster study of phenolic metabolites using docking affinity to soybean LOX-1.

**FIGURE 6:**
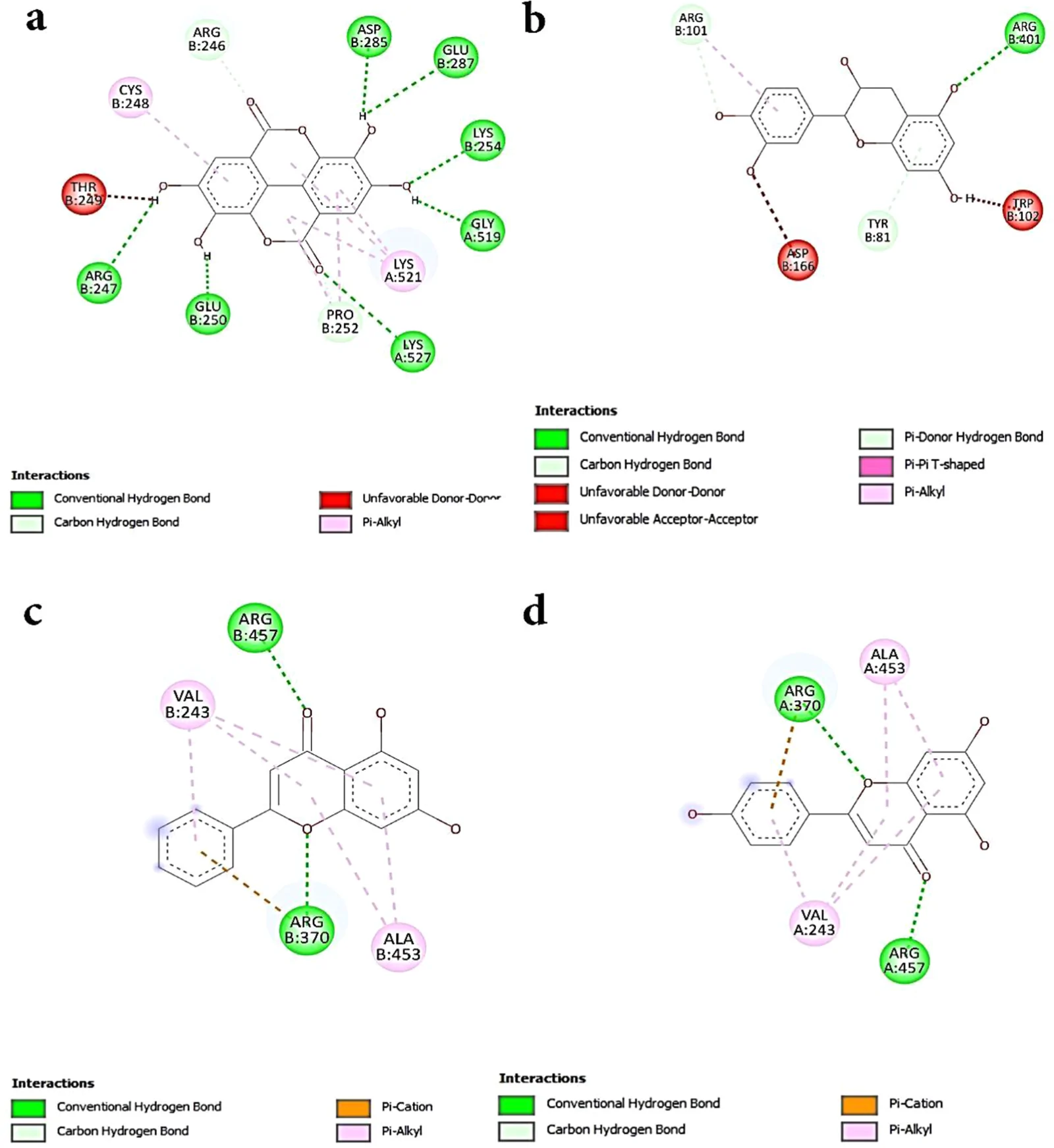
Binding interactions between ellagic acid **(a)**, catechin **(b)**, chrysin **(c)**, and quercetin **(d)** and soybean LOX-1.

**FIGURE 7:**
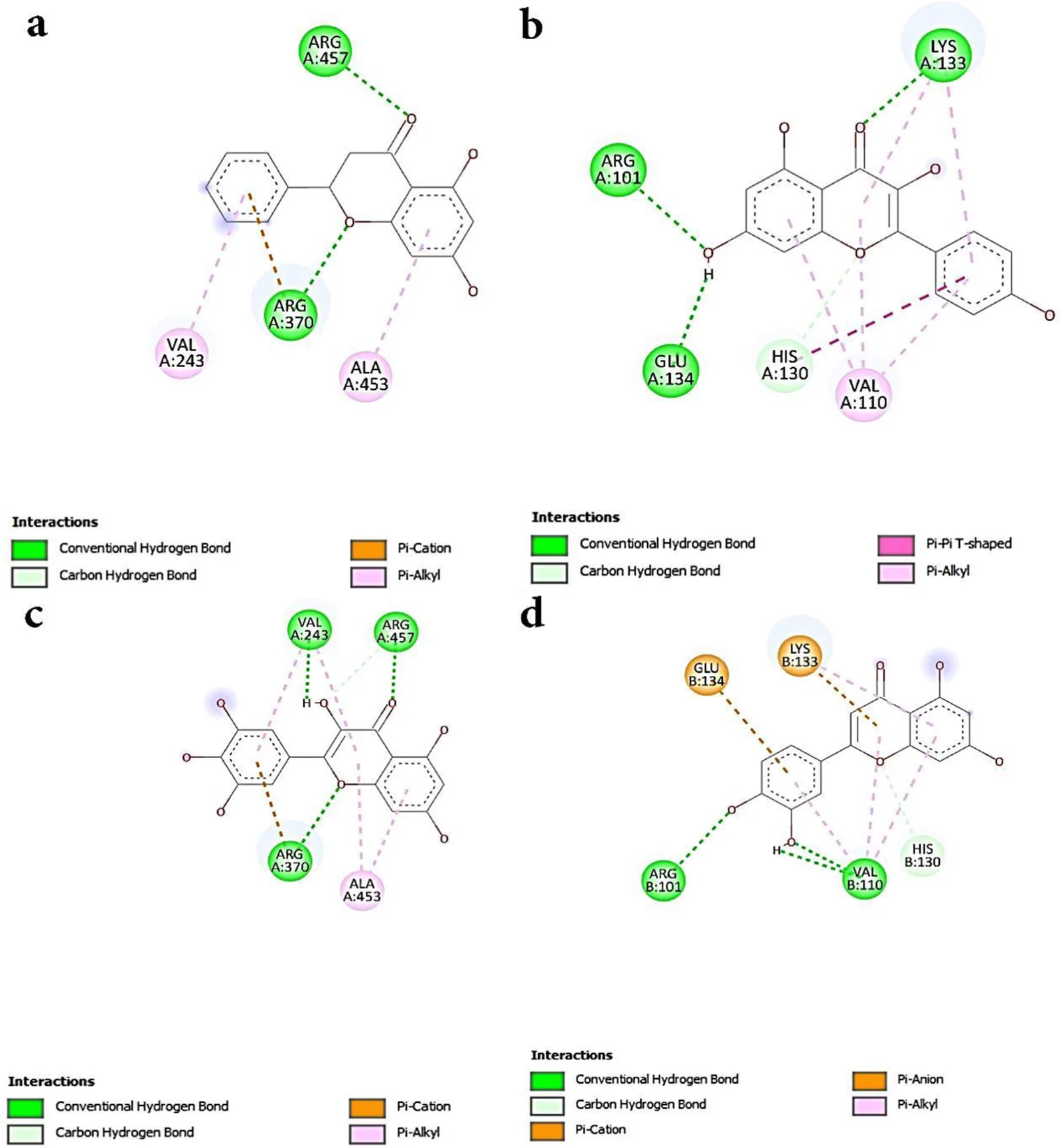
Binding interactions between pinocembrin **(a)**, kaempferol **(b)**, myricetin **(c)**, and luteolin **(d)** and soybean LOX-1.

Molecular docking analysis demonstrated that ellagic acid, catechin, chrysin, and quercetin established several stabilizing interactions within the catalytic cavity of soybean lipoxygenase-1. These metabolites interacted with key amino acid residues through hydrogen bonding, hydrophobic contacts, and π–π interactions. Ellagic acid, chrysin, and quercetin showed particularly strong binding profiles owing to the existence of multiple hydroxyl groups capable of forming stable hydrogen bonds with catalytic residues. Catechin displayed favorable hydrogen-bond and hydrophobic contacts but formed comparatively fewer interactions than ellagic acid, chrysin, and quercetin, which may explain its relatively lower affinity. The interaction network observed for these metabolites suggests efficient stabilization inside the active site of soybean LOX-1 and supports their inhibition against the enzyme. Strong ligand accommodation within the active site of soybean lipoxygenase-1 was also observed for pinocembrin, kaempferol, myricetin, and luteolin through multiple hydrogen bonding and hydrophobic interactions. The binding conformations involved important amino acid residues. Myricetin and luteolin exhibited the strongest interaction patterns, likely because their highly hydroxylated structures enabled the formation of multiple hydrogen bonds with surrounding residues. Kaempferol also demonstrated favorable stabilization within the catalytic cavity, while pinocembrin showed relatively weaker interactions due to its lower hydroxylation degree. These findings indicate that flavonoid structural characteristics and hydroxyl group distribution increase binding affinity toward soybean LOX-1, thereby contributing to the predicted cholesterol gallstone dissolution of phenolic metabolites. These metabolites, particularly flavonoids like quercetin, kaempferol, myricetin, pinocembrin, chrysin, and luteolin, inhibit soybean lipoxygenase-1.

This study has some limitations. The dissolution assay used gallstones from a single patient and compared the extract only with a saline control, without a solvent (vehicle) control or an established litholytic agent such as ursodeoxycholic acid; part of the observed dissolution could therefore reflect nonspecific solubilization rather than a specific pharmacological effect. The results also rest on technical replicates from a small number of gallstones, so the compositional range of clinical gallstones is not captured. The docking used one rigid soybean LOX-1 structure (PDB 3O8Y) and indicates binding tendencies rather than confirmed inhibition, and none of the activities were tested in cells or animals. Follow-up work should add solvent and positive controls to the dissolution assay, dose-response testing in hepatobiliary cell lines, *in vivo* lithogenic-diet models, bioavailability and toxicity profiling of the main phenolics, and molecular dynamics simulations to confirm the stability of the predicted complexes.

## 4 Conclusion

The present investigation found that the methanolic extract of Algerian *C. siliqua* pods is rich in phenolic metabolites. The extract exhibited low IC_50_ values in the DPPH and FRAP analytical descriptors assays, reflecting pronounced free-radical scavenging and ferric ion reduction, suggesting a role in limiting oxidative stress. In addition, MECp showed highly significant *in vitro* cholesterol gallstone-dissolving activity, indicating potential worth exploring for non-surgical cholelithiasis management. Molecular docking indicated stable interactions between the main phenolics and the soybean LOX-1 active site, consistent with an anti-inflammatory mechanism that could limit gallstones formation rather than one that directly explains dissolution. Overall, the polyphenols of *C. siliqua* may have therapeutic potential for pharmacological and nutritional applications. More *in vivo* and toxicity assessments are necessary to validate the clinical potential of *C. siliqua* pod extract for cholesterol gallstone dissolution.

## Data Availability

The raw data supporting the conclusions of this article will be made available by the authors, without undue reservation.
